# Fatty Acid Profiles of Cow’s Milk and Cheese as Affected by Mountain Pasture Type and Concentrate Supplementation

**DOI:** 10.3390/ani9020068

**Published:** 2019-02-22

**Authors:** Mirco Corazzin, Alberto Romanzin, Angela Sepulcri, Maurizio Pinosa, Edi Piasentier, Stefano Bovolenta

**Affiliations:** Department of Agricultural, Food, Environmental and Animal Sciences, University of Udine, 33100 Udine, Italy; mirco.corazzin@uniud.it (M.C.); angela.sepulcri@uniud.it (A.S.); maurizio.pinosa@uniud.it (M.P.); edi.piasentier@uniud.it (E.P.); stefano.bovolenta@uniud.it (S.B.)

**Keywords:** dairy cow, alpine pasture, supplement, milk, cheese, fatty acid

## Abstract

**Simple Summary:**

It is well-known that milk and cheese derived from grazing animals are beneficial for human health. Grazing dairy cows produce milk with high levels of unsaturated fatty acids and conjugated linoleic acid, which are able to reduce cardiovascular diseases and have some anticancer properties. The aim of this trial was to assess the effect of pasture type and concentrate supplementation levels on the fatty acid composition of milk and cheese obtained during summer grazing on mountain pasture. Seventy-two dairy cows, supplemented with 3.0 kg/head/d or 1.5 kg/head/d of energetic concentrate feed in the diet, grazed on a nutritionally rich *Poion alpinae* pasture, and subsequently a nutritionally poor *Seslerion caeruleae* pasture. In milk, the highest concentrate level reduced linolenic acid and total polyunsaturated fatty acids, while the pasture type influenced the monounsaturated fatty acids. In cheeses, these differences were markedly reduced.

**Abstract:**

The aim of this trial was to assess the effect of pasture type and concentrate supplementation on the fatty acids (FA) composition of milk and cheese obtained during summer grazing on mountain pasture. Seventy-two Italian Simmental dairy cows were assigned to two groups that differed by the amount of concentrate supplementation: 3.0 kg/head/d (HIGH) vs. 1.5 kg/head/d (LOW). The dairy cows grazed on a *Poion alpinae* alliance pasture (PAST1), and subsequently they grazed on a *Seslerion caeruleae* alliance pasture (PAST2) for 10 d each. In the last three days of each experimental period, milk samples were collected immediately before each cheese-making event. Cheese samples were collected from each cheese loaf after 60 d of ripening. LOW showed higher *iso*FA, FA intermediates of the ruminal biohydrogenation, C18:3 *c*9,*c*12,*c*15, and total polyunsaturated fatty acid (PUFA) levels than HIGH. The pasture type had a more limited effect on FA composition of milk than concentrate level and was mainly related to monounsaturated fatty acids (MUFA), which were higher in PAST1 than PAST2 (*p* < 0.05). In cheeses, these differences were reduced. The phytanic acid and phytanic isomer ratio (SRR/RRR) in milk were not affected either by supplement level (*p* > 0.05) or by type of pasture (*p* > 0.05). Increasing the concentrate offered to dairy cows from 1.5 to 3.0 kg/d did not markedly influence the level of PUFA in cheeses produced during summer grazing on high mountain pasture.

## 1. Introduction

In Europe, from 2010 to 2013, the area of permanent grassland and meadows was reduced by 2%, while the number of holdings with permanent grassland and meadows was reduced by 9% [1]. These reductions have been even more pronounced in less favored areas, such as high mountains, where large areas of grasslands have been abandoned [2]. Salvador et al. [3] reported that the number of meadows and pastures, and the number of farms have decreased by over 25% and 60% in the Italian Alps, respectively. In mountain areas, grasslands allow for the subsistence of dairy farms by providing much of the ruminants’ feed. Mountain dairy farms are widely recognized as providing a so-called ‘ecosystem service’ to society [4]. Indeed, they have a role in carbon sequestration, in soil fertility, in cultural heritage maintenance, and in fire hazard prevention [5]. Moreover, especially during the summer, mountain and Alpine farms are increasingly linked to the development of tourism in the area, thanks to the unspoiled environment that they can offer to the tourist [6].

In mountain areas, dairy cows are kept indoors all year with the exception of the summer months, when the cows are allowed to graze. During the grazing season, dairy cows are usually moved through different pastures to take full advantage of the available territory following the vegetation gradient and the so-called ‘vertical transhumance’ animal management strategy. Moreover, the use of pasture is considered as a tool to increase the added value of summer dairy products obtained from mountain farms [7,8]. Indeed, it is well-known that dairy cows fed pasture-based diets produce milk richer in unsaturated fatty acids than cows fed total mixed ration or hay-based diets [7,9,10,11]. Several studies have shown that these fatty acids (FA) are favorable to human health. In particular, polyunsaturated fatty acids (PUFA) are able to decrease cardio-vascular disease risk [12] and, in animal models, conjugated linoleic acids (CLA) have been shown to be involved in anti-carcinogenic, immunomodulatory, and anti-diabetic activities [13]. Over the years, these findings have allowed dairy products to acquire a healthy image, increasing consumer interest. From this point of view, some markers, such as phytanic acid and the ratio between its two isomers—3S, 7R, 11R, 15-phytanic acid (SRR) and 3R, 7R, 11R, 15-phytanic acid (RRR)—were proposed to distinguish dairy products on the basis of the concentrate or fresh herbage offered to animals [14]. The effect of pasture on the FA composition of milk and cheese is highly variable depending on many factors, such as the amount of herbage intake, botanical and chemical composition and vegetative stage of the pasture, and concentrate feed supply in animals’ diets [10,15,16,17]. Indeed, in recent decades, the intensification process of husbandry systems has caused an increase in the nutritional requirements of lactating cows. Fresh forage is no longer enough to ensure the nutritional requirements and welfare of the main cosmopolitan breeds of grazing cows. Lactating cows especially must receive adequate supplementation to reduce the loss of milk production and body condition. The concentrate level in the diet can influence not only the FA composition of dairy products, but also the productive performance, eating behavior, and ruminal activity of grazing cows [18,19].

This study aimed to assess the effect of pasture type and concentrate supplementation on the FA composition of milk and cheese obtained during summer grazing. These findings could be useful to enhance the summer dairy products and to reduce the abandonment of farms in mountain area.

## 2. Materials and Methods 

The trial was carried out in accordance with EU Directive 2010/63/EU; it complied with the Italian legislation on animal care (DL n. 26, 4 March 2014), and adhered to the internal rules of the University of Udine.

### 2.1. Experimental Design and Samples Collection

Seventy-two Italian Simmental dairy cows grazing day and night on high mountain pasture (Malga Montasio, Italy; lat 46°24′45″ N, long 13°25′53″ E; 1500–1800 m asl) were assigned to two homogeneous experimental groups according to their productive performance recorded after a preliminary period of two weeks for milk yield (16.9 ± 3.3 kg/d), stage of lactation (192.8 ± 64.0 days in milk (DIM)), fat (3.94 ± 0.25%), protein (3.29 ± 0.14%), lactose (4.71 ± 0.12%), and somatic cell count (SCC) (130,000 ± 47,000 cells/mL). The groups differed for amount of concentrate supplementation: 3.0 kg/head/d (HIGH) vs. 1.5 kg/head/d (LOW). The concentrate was mainly based on maize, barley, beet pulp, and soy, and its chemical composition and fatty acid profile are reported in Table 1. The dairy cows grazed on a nutritionally rich pasture (*Poion alpinae* alliance pasture, 1500 m asl; PAST1) for 10 d, and after another adaptation period of two weeks, they grazed on a nutritionally poor pasture (*Seslerion caeruleae* alliance pasture, 1700 m asl; PAST2) for 10 d. PAST 1 was mainly composed of 66.5% *Poaceae* and *Cyperaceae*, 7.4% *Ranunculaceae*, and 2.8% *Fabaceae*, while PAST2 was mainly composed of 61.8% *Poaceae* and *Cyperaceae*, 8.5% *Asteraceae*, 5.4% *Fabaceae*, and 3.3% *Rosaceae*. Both pastures were grazed at the flowering stage of *Poaceae* with shepherd-guided grazing. Concentrate was offered to animals twice daily in the milking parlor. More details are reported in Bovolenta et al. [20]. For three days distributed equally over the grazing period, samples of the herbage, selected by four cows randomly chosen in each experimental group, were collected following the hand-plucking technique [21], which is manual collection of the herbage simulating animal bite size. These samples were bulked within day, and treatment and the proximate and FA analysis were carried out. The cheese-making was performed using whole raw milk, in accordance with PDO Montasio product specifications and as reported in Bovolenta et al. [20], in the three last days for each experimental period and for each experimental group, mixing evening and morning milk. A total of 12 loaves of cheese were produced. For the chemical analysis, bulked milk samples were collected immediately before each cheese-making, while cheese samples were collected from each loaf after 60 d of ripening and after removing 10 mm of rind.

### 2.2. Chemical Analyses 

The herbage and concentrate samples were dried in a forced draft oven at 65 °C for 48 h and analyzed for crude protein (CP) and ether extract (EE) according to Association of Official Agricultural Chemists (AOAC) [22], and for neutral detergent fiber (NDF) according to Goering and Van Soest [23].

On milk samples, the following determinations were carried out: fat, protein, lactose [24], urea [22], and somatic cell count (SCC; Foss-o-Matic, Foss Electric, Hillerod, Denmark). SCC data was analyzed as: somatic cell score (SCS) = log_2_ (SCC/100,000) + 3 [25]. 

The cheese samples were analyzed for fat and protein according to AOAC [22]. 

Fatty acids of herbage and concentrate samples were determined as methyl esters, extracted by chloroform:methanol (2:1, *v*/*v*) [26], and esterified using methanolic hydrogen chloride [27]. Fatty acid methyl esters were separated in a gas chromatograph (HRGC 5300, Carlo Erba, Cornaredo, Italy) with split injection (ratio 1:50), flame ionization detector (FID), and ChemStation software (Hewlett Packard 3365, Avondale, PA, USA) for chromatogram integration. The capillary column was an SP 2380 capillary column (60 m × 0.25 μm × 0.25 mm; Supelco, Oakville, ON, USA), with a temperature program from 160 to 260 °C. Injector temperature was set at 250 °C and the detector was set at 260 °C.

Fat was extracted from milk following the method described by Povolo et al. [28] in order to obtain the fat without using solvents. Fat extraction from cheese, conversely, was performed according to the modified Folch’s technique [29].

Either way, the lipids were then esterificated in accordance with the method described by Bannon et al. [30], with modifications reported by Prandini et al. [31].

Fatty acid methyl esters were detected using a GCMS Saturn 2100T (Agilent Technologies, Italy S.p.a., Cernusco sul Naviglio, Italy) equipped with an HP-88 (Agilent Technologies, Santa Clara, CA, USA) capillary column (100 m length, 0.25 mm i.d, 0.20 μm film thickness). Split injection (1 µl) was adopted (ratio 1:50) and helium was used as a carrier gas at a flow rate of 1.2 mL/min. Oven temperature was held at 50 °C for 5 min, programmed to 160 °C at a rate of 10 °C/min, held at 160 °C for 10 min, programmed to 180 °C at 0.3 °C/min, held to 180 °C for 5 min, and then increased to 240 °C at 20 °C/min, and held at 240 °C for 10 min. The transfer line was held at 240 °C and the mass spectroscopy source at 200 °C. Acquisition was performed in electron ionization mode (70 eV) at 0.4 scan/s, and the mass range used was 10–400 m/z. The identification of the compound was made by using the NIST library (National Institute of Standards and Technology—NIST, Gaithersburg, MD, USA), the MS data of literature, and when available, the injection of authentic standards. Fatty acid composition was expressed as g/100 g of total fatty acids of milk and cheese, respectively.

### 2.3. Statistical Analysis

Data were analyzed using R software [32]. The effect of pasture type (PAST1 vs. PAST2) and concentrate level (HIGH vs. LOW) on herbage chemical characteristics was analyzed by a general linear model that considered pasture type and concentrate level as fixed effects. The milk and cheese chemical composition was subjected to a hierarchical model as reported by Bovolenta et al. [20] with pasture type and supplement level considered as fixed factors and the cheese-making day nested within the pasture treated as a random factor. 

## 3. Results and Discussion

Chemical composition and fatty acid (FA) profiles of the herbage selected by cows and of the concentrate offered are reported in Table 1. 

The supplement level did not influence the chemical composition or the FA profile of the herbage selected by the animals (*p* > 0.05). PAST1 showed higher CP (*p* < 0.01) and lower dry matter (DM, *p* < 0.01), ash (*p* < 0.05), and EE (*p* < 0.01) concentrations than PAST2. The differences between the FA profiles were limited to C20:4 c5,c8,c11,c14, the highest in PAST2 (*p* < 0.05). Considering that the two experimental pastures were grazed at the same phenological stage, these differences may be due to their different botanical composition, as explained by Bovolenta et al. [33].

The pasture type and the supplement level did not influence (*p* > 0.05) the chemical composition of milk in terms of fat (3.99 ± 0.043%), protein (3.28 ± 0.008%), and SCS (4.03 ± 0.166 units), and of cheese in terms of DM (66.6 ± 0.206%), fat (53.2 ± 0.364% DM), protein (40.5 ± 0.404 % DM). The milk composition was similar to that reported by Romanzin et al. [18], who considered milk from Italian Simmental dairy cows grazing on mountain pasture, and by Coppa et al. [34] who considered milk from Piemontese and Simmental breeds. The urea in milk was higher in PAST2 than in PAST1 (23.3 vs. 20.3, *p* < 0.01) and higher in LOW than in HIGH groups (22.9 vs. 20.7, *p* < 0.01), although its average value was within the range of normality of 15–30 mg/dL proposed by Bendelja et al. [35]. The average SCS observed corresponded to a value of about 204,000 cells/mL which was slightly higher that the threshold, 200,000 cells/mL, proposed for detecting animals with healthy mammary glands [36,37]. However, it was lower than the value of 308,000 cells/mL reviewed by Corazzin et al. [38] and recorded in a wide survey involving a mountain dairy farm. The cheese composition was similar to that reported by Romanzin et al. [7] and Bovolenta et al. [39] in cheeses produced with a very similar production process.

The FA composition of experimental milk is shown in Table 2. 

Considering the saturated FA of milk, the concentrations of C14:0 (*p* < 0.05), *iso*-C14:0 (*p* < 0.01), *iso*-C15:0 (*p* < 0.01), C15:0 (*p* < 0.01), *iso*-C16:0 (*p* < 0.01), *ante-iso*-C17:0 (*p* < 0.01), C16:0 (*p* < 0.05), C20:0 (*p* < 0.05) were higher in LOW than HIGH milk; instead, the C18:0 (*p* < 0.01) level was highest in milk from the HIGH group. Moreover, LOW tended to have higher C17:0 (*p* < 0.10), and *iso*-C18:0 (*p* < 0.10) than the HIGH group. Vlaeminck et al. [42] explained that *iso*-C14:0, *iso*-C15:0, C15:0, *iso*-C16:0 are constituents of the bacterial membrane, and that their variation can reflect variations in the bacterial ruminal population. Indeed, the same authors found that cellulolytic bacteria membranes are particularly rich in *iso*-FA. Therefore, we supposed that the animals from the experimental groups had different ruminal bacterial populations, with the LOW having higher cellulolytic ruminal bacteria than the HIGH group. This hypothesis reflects the probable highest forage/concentrate ratio of the diet of the LOW group. We had to take into account that linear odd-chain FA, and *ante-iso*-FA can also derive from mammary gland de-novo synthesis, and that the C17:1/C17:0 is also regulated by the action of the Δ9 desaturase enzyme [42]. Although Kilcawley et al. [43] argued that higher herbage intake can inhibit de-novo FA synthesis, the effect of concentrate on mammary gland enzyme activity or expression seems to be limited. Indeed, the level of short chain FAs, that are indicators of FA synthesis [44], were similar between experimental groups (*p* > 0.10) with the only exception being C14:0, which was highest in the LOW group (*p* < 0.05). Additionally, the C17:1/C17:0 ratio was similar between experimental groups (0.245 ± 0.011; *p* > 0.05), reinforcing the hypothesis that the differences observed for the saturated FA previously reported were due to a different selection of ruminal bacterial populations.

Considering the unsaturated FA of milk, the concentrations of C17:1 (*p* < 0.01), C18:1 *t*11 (*p* < 0.01), CLA (*p* < 0.05), C18:2 *t*11,*c*15 (*p* < 0.01), C18:3 *c*9,*c*12,*c*15 (*p* < 0.01), and C20:4 *c*5,*c*8,*c*11,*c*14 (*p* < 0.01) were higher in LOW than HIGH milk; instead, the C18:1 *c*9 (*p* < 0.01), C18:1 *c*11 (*p* < 0.01), and C18:1 *c*12 (*p* < 0.01) levels were highest in milk from the HIGH group. Moreover, LOW milk tended to have higher C16:1 *c*7 (*p* < 0.10) and the sum of C18: *t*12/*t*13/*t*14 isomers (*p* < 0.10), and lower C18:1 *t*6-8-9-10 (*p* < 0.10) than the HIGH group. It is accepted that C18:3 *c*9,*c*12,*c*15 and C18:2 *c*9,*c*12 originated in the feed. The herbage ingested contained more than 40% of the total FA of C18:3 *c*9,*c*12,*c*15; in particular, the level of this FA was 1.17 g/100 g DM and 0.09 g/100 g DM in herbage and concentrate, respectively. Therefore, it is reasonable that the LOW group, fed with a lower level of concentrate, ingested a higher amount of herbage, explaining the differences observed in the concentration of C18:3 *c*9,*c*12,*c*15. Conversely, it seems that the differences of C18:2 *c*9,*c*12 in the two diets were not enough to generate variations in this FA in the milk of the experimental groups. Indeed, the level of C18:2 *c*9,*c*12 was 0.56 g/100 g DM and 1.50 g/100 g DM in herbage and concentrate, respectively. C18:1 *t*11 and C18:2 *t*11,*c*15 are intermediates of the ruminal biohydrogenation of the dietary unsaturated FA, and, therefore, it can be speculated that the ruminal biohydrogenation was higher in animals receiving a lower level of concentrate in accordance with Khanal et al. [45] and Bovolenta et al. [46]. Additionally, Rego et al. [47] showed that increasing the fresh herbage intake by animals, also led to increased levels of C18:1 *t*11, CLA, and C18:3 *c*9,*c*12,*c*15. It is interesting to note that the C18:0 level, a final product of the ruminal biohydrogenation of dietary FA, was higher in the HIGH than LOW group. Taking into account our results, it could be hypothesized that the final step of rumen biohydrogenation was inhibited in the LOW group. Dewanckele et al. [48] explained that diets rich in PUFA can inhibit the last step of rumen biohydrogenation by modifying the rumen bacteria population, resulting in increased intermediate products, as observed also in the present paper. Since C18:1 *c*9 can derive from the desaturation of C18:0, the difference observed in this FA could be due to the different levels of C18:0 between experimental groups.

Considering the groups of FA, the level of PUFA (*p* < 0.01) was higher, while the level of MUFA was lower (*p* < 0.01) in LOW than HIGH group. Romanzin et al. [7], comparing the FA profile of milk from dairy cows grazing on mountain pasture or fed a hay-based diet, showed that the herbage intake increased the level both of PUFA and MUFA. However, in agreement with the present study, Marín et al. [49] showed that by reducing concentrate supplementation to grazing cows from 6–8 kg/d to 1–2 kg/d, the level of milk MUFA decreased.

The pasture type had a more limited effect on FA composition of milk than concentrate levels in animals’ diets. Indeed, PAST1 showed higher levels of C10:1 (*p* < 0.01), C12:0 (*p* < 0.05), C16:0 (*p* < 0.01), C18:1 *c*14/*t*16 (*p* < 0.01), and MUFA (*p* < 0.01), but lower levels of *iso*-C14:0 (*p* < 0.01), *iso*-C15:0 (*p* < 0.01), *ante-iso*-C15:0 (*p* < 0.05), *iso*-C16:0 (*p* < 0.01), *ante-iso*-C17:0 (*p* < 0.01), C17:0 (*p* < 0.01), *iso*-C18:0 (*p* < 0.01), C18:1 *c*12 (*p* < 0.01), C18:1 *t* (*p* < 0.05), and C20:0 (*p* < 0.01) than the PAST2 group. Despite not being measured, the difference in the herbage intake by animals between PAST1 and PAST2 can be considered very small following the INRA standard [50]. As for the different level of concentrate supplementation and taking into account the results about *iso*-FA, *ante-iso*-C15:0, and *ante-iso*-C17:0, we can speculate that the different pasture type was able to influence the ruminal bacteria population. Conversely, in the present study, since only C12:0 of the short chain FAs was different between pastures, the effect of pasture type on mammary de novo synthesis of FA was not evident. Similarly, taking into account that only C18:1 *t* and C18:1 *c*12 were different between experimental groups, the effect of pasture type on ruminal biohydrogenation of dietary FA was also not evident. In the literature, the effect of pasture type on milk FA is highly variable. Khiosa-Ard et al. [51] found that the level of C18:3 *c*9,*c*12,*c*15 in milk was influenced by the botanical family of plants ingested by animals, which is not in agreement with the results of the present study. Falchero et al. [52], comparing two alpine pastures, showed differences only in the level of C15:0 and C16:0 of milk. Gorlier et al. [53] found that the pasture type was not able to influence the FA profile of milk; at the same time, they showed that the presence of the botanical family of *Poaceae* was correlated positively with MUFA and negatively with C18:2 *c*9,*c*12 levels in milk. Conversely, Collomb et al. [54], with regard to *Poaceae*, reported a significant positive correlation with SFA, a negative significant correlation with PUFA, and no correlation with MUFA. The Alpine pastures are characterized by a high botanical diversity, and different plants can contain different levels of secondary metabolites, such as polyphenol oxidase and tannins, that can influence the metabolism of FA [55]. Therefore, not only the plant family, but also the single plant species of the pasture can influence the FA composition [54,56].

Phytanic acid and its isomers’ SRR/RRR ratio in milk was not affected either by supplement level (*p* > 0.05) or type of pasture (*p* > 0.05). Only the LOW group tended to have higher phytanic acid content than the HIGH group (*p* < 0.10). Phytanic acid cannot be synthetized by dairy cows and has a dietary origin deriving from the metabolism of chlorophyll in the rumen, while the SRR/RRR diastereomer ratio depends on the microbial flora present in the rumen as a consequence of the animal’s diet [14,57]. Therefore, phytanic acid, and its SRR/RRR diastereomer ratio were proposed as markers for authentication of organic milk, and consequently, to assess the fresh herbage feeding of dairy cows [14]. However, Che et al. [58] observed a significant and high correlation between the SRR/RRR ratio and intake of grazed clovers, but these authors failed to find a correlation between the SRR/RRR ratio and intake of grazed grass by cows. In the present trial, the different amounts of concentrate offered between experimental groups was probably too low to provide differences in the phytanic acid level and SRR/RRR ratio in milk. This statement seems to be supported by the fact that Vetter and Schröder [57] indicated a threshold of 0.20% of phytanic acid for distinguishing organic from conventional dairy products, and in the present trial, all the experimental milk showed a phytanic acid level higher than 0.25%.

Many studies have reported that SFA, and C12:0, C14:0, and C16:0 in particular, have hypercholesterolemic properties and favor the increase of blood low density lipoprotein [59,60]. Conversely, PUFA, and C18:3 *c*9,*c*12,*c*15 in particular, have beneficial effects for human health, such as reduction of plasma triacylglycerol and plasma pressure [59]. In the present trial, the LOW group showed levels of PUFA and C18:3 *c*9,*c*12,*c*15 that were 15% and 12% higher, respectively, than those shown by the HIGH group. Moreover, the milk produced from cows grazing on *Seslerion caeruleae* alliance pasture (PAST2 group) showed a level of C12:0 and C16:0 around 1% lower than those produced from cows grazing on *Poion alpinae* alliance pasture (PAST1 group). Therefore, LOW and PAST2 had a more beneficial FA profile of milk than HIGH and PAST1 groups, respectively. 

The FA composition of experimental cheeses is showed in Table 3. 

The concentrations of *iso*-C15:0 (*p* < 0.05), *iso*-C17:0 (*p* < 0.01), C17:1 (*p* < 0.01), C18:0 (*p* < 0.01), C18:1 *t*11 (*p* < 0.01), C18:1 *c*9 (*p* < 0.01), C18:1 *t*15 (*p* < 0.05), and C18:3 *c*9,*c*12,*c*15 (*p* < 0.01) were higher in LOW than HIGH cheese; instead, the C4:0 (*p* < 0.01), C8:0 (*p* < 0.01), C10:0 (*p* < 0.01), C12:0 (*p* < 0.01), C13:0 (*p* < 0.01), C18:1 *c*12 (*p* < 0.05), and C20:0 (*p* < 0.01) levels were highest in cheese from the HIGH group. Coppa et al. [61] observed that in cheeses ripened 12 weeks, the effect of the cheese-making process had a very small effect on the FA profile. Conversely, in the present paper, despite the differences in iso-C15:0, C17:1, C18:1 *t*11, and C18:3 *c*9,*c*12,*c*15 reflected in those of milk, some differences in FA composition in milk and cheese were found. These differences could be due to the lipolysis process that cheese underwent during ripening. From this point of view, one of the most important lipolytic agents is the lipoprotein lipase (LPL) derived from the milk and that is inactivated after pasteurization. This enzyme preferentially acts towards short chain fatty acids, C4-C12 [62,63]. The results of the present paper could be explained if we suppose that the different herbage intake could have influenced the LPL expression and/or activity, as previously reported in cattle by Palin et al. [64] and Corazzin et al. [65]. Unlike the results related to supplement levels, as expected the differences between cheese FA composition due to pasture type mainly reflected those observed in milk. Indeed, the cheese produced from the PAST1 group had higher C10:1 (*p* < 0.01), and C18:1 *t*15 (*p* < 0.01), but lower *iso*-C14:0 (*p* < 0.01), *iso*-C15:0 (*p* < 0.01), *ante-iso*-C15:0 (*p* < 0.05), *iso*-C16:0 (*p* < 0.01), C15:0 (*p* < 0.01), *iso*-C16:0 (*p* < 0.01), C17:0 (*p* < 0.01), C18:1 *c*12 (*p* < 0.01), C18:2 CLA (*p* < 0.05), and C20:0 (*p* < 0.01) levels than those from the PAST2 group.

Considering the categories of FA, it is interesting to note that the cheese-making process and the ripening period reduced the differences between experimental groups, especially considering the supplement level factor. Indeed, the cheese of the HIGH group showed similar SFA, MUFA, and PUFA levels to those of the LOW group. Additionally, the total concentration of these categories of FA was similar between cheese and milk independent of the experimental factor. The average total amounts of SFA, MUFA, and PUFA were 66.9, 27, and 6.1% respectively, which were similar to those reported by Romanzin et al. [7] in the same type of product, Montasio PDO cheese, which were 64.1, 32.2, and 3.7% respectively.

As for milk, phytanic acid level and its SRR/RRR diastereomer ratio were similar between experimental groups (*p* > 0.05).

## 4. Conclusions

From the milk FA profile point of view, increasing the concentrate offered to cows decreased the level of FA beneficial for human health, such as PUFA and C18:3 *c*9,*c*12,*c*15, as well as some FA intermediates of rumen biohydrogenation. Conversely, the effect of the two pasture types considered was more limited. 

The differences in PUFA levels in all the experimental cheeses, probably because of the cheese-making process and the ripening period, were reduced. Finally, we can conclude that the increase in the concentrate offered to dairy cows from 1.5 to 3.0 kg/d, which can have a nutritional role, does not markedly influence the level of SFA, MUFA, and PUFA in cheeses produced during summer grazing on different high mountain pastures.

## Figures and Tables

**Table 1:** Chemical composition and fatty acid profiles of the herbage selected by cows as affected by pasture type and supplement level (*n* = 12), and of the concentrate offered.

Items	Herbage Selected							Concentrate Offered
	Pasture Type		Supplement Level		SEM	*p*–Value		
	PAST1	PAST2	HIGH	LOW		P	S	
Chemical composition, g/100 g DM								
DM	26.9	32.2	29.3	29.8	0.31	<0.001	0.402	87.9
CP	12.9	11.2	12.2	11.9	0.12	<0.001	0.173	14.7
EE	2.5	2.8	2.7	2.7	0.03	0.001	0.665	2.7
NDF	53.8	53.9	53.1	54.6	0.56	0.916	0.221	-
Fatty acid profile, g/100 g of total fatty acids								
C10:0	0.60	0.59	0.57	0.62	0.027	0.868	0.366	-
C12:0	0.64	0.52	0.67	0.49	0.066	0.361	0.227	-
C14:0	1.56	1.33	1.67	1.23	0.216	0.615	0.333	0.06
C16:0	18.16	17.38	18.21	17.33	0.334	0.274	0.221	13.70
C16:1	0.92	0.87	0.88	0.91	0.047	0.659	0.748	-
C17:0	0.47	0.42	0.48	0.41	0.033	0.499	0.296	-
C18:0	3.63	2.93	3.51	3.05	0.286	0.252	0.433	5.99
C18:1 *c*9	8.12	8.69	8.26	8.54	0.428	0.523	0.753	21.56
C18:2 *c*9,*c*12	19.77	21.76	20.68	20.85	0.465	0.061	0.860	55.42
C18:3 *c*6,*c*9,*c*12	0.88	0.98	0.90	0.95	0.055	0.405	0.681	-
C18:3 *c*9,*c*12,*c*15	44.06	42.65	42.77	43.93	1.030	0.509	0.587	3.27
C20:3 *c*11,*c*14*,c17*	0.54	0.69	0.62	0.61	0.052	0.183	0.899	-
C20:4 *c*5,*c*8,*c*11,*c*14	0.66	1.20	0.77	1.08	0.089	0.014	0.113	-

SEM: standard error of the mean; P: pasture type; S: supplement level; DM: dry matter; CP: crude protein; EE: ether extract; NDF: neutral detergent fiber; *c*: cis.

**Table 2:** Fatty acid composition (g/100 g of total fatty acids) of milk as affected by pasture type and supplement level; *n* = 12 ^1^.

Fatty Acid	Pasture Type		Supplement Level		SEM	*p*-Value	
	PAST1	PAST2	HIGH	LOW		P	S
C4:0	4.66	4.54	4.62	4.58	0.036	0.148	0.531
C6:0	2.71	2.64	2.69	2.66	0.025	0.174	0.519
C8:0	1.46	1.42	1.45	1.43	0.014	0.183	0.541
C10:0	2.83	2.78	2.81	2.80	0.013	0.101	0.669
C10:1	0.22	0.19	0.20	0.21	0.003	<0.001	0.798
C12:0	2.97	2.93	2.95	2.96	0.006	0.013	0.588
C13:0	0.16	0.14	0.16	0.14	0.008	0.343	0.343
*iso*-C14:0	0.20	0.23	0.20	0.23	0.003	0.002	0.002
C14:0	10.00	9.95	9.93	10.02	0.018	0.137	0.048
*iso*-C15:0	0.40	0.47	0.41	0.46	0.004	<0.001	<0.001
*anteiso*-C15:0	0.88	0.98	0.91	0.96	0.016	0.010	0.158
C14:1 *c*9	0.69	0.63	0.66	0.66	0.022	0.178	0.940
C15:0	1.39	1.45	1.37	1.48	0.014	0.064	0.003
*iso*-C16:0	0.41	0.49	0.43	0.47	0.005	<0.001	0.007
C16:0	24.97	24.75	24.79	24.93	0.024	0.001	0.021
C16:1 *c*7	0.23	0.24	0.22	0.24	0.005	0.307	0.058
*iso*-C17:0	0.55	0.56	0.55	0.56	0.003	0.624	0.163
C16:1 *c*9	1.38	1.48	1.40	1.46	0.033	0.192	0.414
*anteiso*-C17:0	0.72	0.80	0.73	0.80	0.007	<0.001	0.001
Phytanic	0.31	0.33	0.30	0.34	0.009	0.206	0.061
C17:0	0.87	1.09	0.95	1.01	0.015	<0.001	0.057
*iso*-C18:0	0.08	0.10	0.09	0.10	0.003	0.003	0.081
C17:1	0.24	0.24	0.25	0.26	0.001	0.161	<0.001
C18:0	10.89	10.82	11.05	10.66	0.017	0.076	<0.001
C18:1 *t*6-8-9-10	0.34	0.29	0.43	0.21	0.055	0.630	0.079
C18:1 *t*11	3.03	3.01	2.97	3.07	0.005	0.086	<0.001
C18:1 *c*9	19.38	19.26	19.76	18.88	0.030	0.075	<0.001
C18:1 *t*15	0.22	0.15	0.17	0.20	0.017	0.104	0.360
C18:1 *c*11	0.50	0.59	0.67	0.42	0.034	0.106	0.007
C18:1 *c*12	0.20	0.25	0.25	0.20	0.005	0.001	0.002
C18:1 *c*14 + *t*16	0.55	0.19	0.37	0.37	0.041	0.002	0.945
C18:1 *t*12 + *t*13 + *t*14	0.04	0.13	0.05	0.12	0.016	0.025	0.074
C18:2 *t*11,*c*15	0.81	0.79	0.68	0.92	0.028	0.792	0.002
C18:2 *c*9,*c*12	2.00	2.02	1.90	2.13	0.063	0.948	0.104
C20:0	0.23	0.35	0.26	0.31	0.011	<0.001	0.048
C18:3 *c*9,*c*12,*c*15	1.09	1.08	1.03	1.15	0.002	0.096	<0.001
C18:2 *c*9,*t*11	1.36	1.35	1.35	1.36	0.002	0.082	0.039
C18:2 CLA ^2^	0.46	0.64	0.44	0.66	0.042	0.059	0.029
C20:4 *c*5,*c*8,*c*11,*c*14	0.07	0.08	0.05	0.10	0.003	0.133	<0.001
Phytanic SRR/RRR ^3^	0.428	0.433	0.401	0.460	0.019	0.893	0.158
SFA	66.86	66.98	66.82	67.02	0.073	0.433	0.219
MUFA	27.13	26.79	27.51	26.41	0.041	0.003	<0.001
PUFA	6.02	6.23	5.67	6.58	0.052	0.066	<0.001

SEM: standard error of the mean; P: pasture type; S: supplement level; CLA: conjugated linoleic acid; SFA: saturated FA; MUFA: monounsaturated FA; PUFA: polyunsaturated FA; *c*: cis; *t*: trans. ^1^ Fatty acids detected at <0.1% of total lipids are not reported; ^2^ This peak can include *t*7,*c*9 and *t*8,*c*10 according to Collomb et al. [40] and Kramer et al. [41], C18:2 *c*9,*t*11 is excluded; ^3^ Ratio between diastereomers of phytanic, 3S,7R,11R,15 and 3R,7R,11R,15.

**Table 3:** Fatty acid composition (g/100 g of total fatty acids) of cheese as affected by pasture type and supplement level; *n* = 12 ^1^.

Fatty Acid	Pasture Type		Supplement Level		SEM	*p*-Value	
	PAST1	PAST2	HIGH	LOW		P	S
C4:0	4.61	4.60	4.65	4.56	0.013	0.737	0.007
C6:0	3.26	2.68	3.34	2.61	0.282	0.330	0.229
C8:0	1.46	1.45	1.52	1.39	0.004	0.775	<0.001
C10:0	2.93	2.93	3.06	2.81	0.009	0.775	<0.001
C10:1	0.21	0.17	0.18	0.20	0.008	0.043	0.185
C12:0	3.13	3.12	3.23	3.02	0.009	0.766	<0.001
C13:0	0.17	0.16	0.17	0.16	0.001	0.755	<0.001
*iso*-C14:0	0.19	0.22	0.20	0.21	0.002	<0.001	0.144
C14:0	10.50	10.47	10.55	10.42	0.031	0.732	0.074
*iso*-C15:0	0.38	0.45	0.41	0.42	0.004	<0.001	0.049
*anteiso*-C15:0	0.77	0.88	0.82	0.83	0.012	0.002	0.867
C14:1 *c*9	0.75	0.70	0.72	0.73	0.012	0.050	0.712
C15:0	1.34	1.41	1.36	1.38	0.012	0.020	0.555
*iso*-C16:0	0.38	0.44	0.42	0.40	0.008	0.007	0.332
C16:0	24.86	24.81	24.88	24.78	0.072	0.728	0.515
C16:1 *c*7	0.19	0.20	0.19	0.20	0.007	0.712	0.332
*iso*-C17:0	0.58	0.58	0.57	0.59	0.002	0.699	<0.001
C16:1 *c*9	1.34	1.37	1.34	1.37	0.022	0.528	0.505
*anteiso*-C17:0	0.68	0.56	0.52	0.72	0.058	0.300	0.135
Phytanic	0.40	0.38	0.36	0.42	0.019	0.609	0.158
C17:0	0.85	0.99	0.93	0.91	0.011	<0.001	0.488
*iso*-C18:0	0.10	0.09	0.10	0.09	0.004	0.790	0.593
C17:1	0.25	0.25	0.24	0.25	0.001	0.696	<0.001
C18:0	10.44	10.42	9.94	10.92	0.030	0.670	<0.001
C18:1 *t*6-8-9-10	0.43	0.48	0.46	0.46	0.025	0.352	0.904
C18:1 *t*11	2.99	2.98	2.94	3.03	0.009	0.708	0.001
C18:1 *c*9	19.47	19.43	19.14	19.76	0.056	0.707	<0.001
C18:1 *t*15	0.16	0.05	0.05	0.15	0.021	0.041	0.044
C18:1 *c*11	0.54	0.49	0.59	0.44	0.087	0.801	0.431
C18:1 *c*12	0.11	0.37	0.36	0.12	0.046	0.021	0.025
C18:1 *c*13	0.22	0.16	0.14	0.23	0.045	0.527	0.338
C18:1 *c*14 + *t*16	0.21	0.10	0.21	0.09	0.061	0.414	0.354
C18:1 *t*12 + *t*13 + *t*14	0.27	0.36	0.37	0.26	0.085	0.589	0.546
C18:2 *t*11,*c*15	0.69	0.74	0.70	0.73	0.020	0.242	0.377
C18:2 *c*9,*c*12	2.06	2.04	2.06	2.06	0.025	0.788	0.978
C20:0	0.10	0.21	0.20	0.11	0.014	0.004	0.009
C18:3 *c*9,*c*12,*c*15	0.98	0.98	0.95	1.00	0.003	0.696	<0.001
C18:2 *c*9,*t*11	1.34	1.34	1.34	1.34	0.004	0.724	0.655
C18:2 CLA ^2^	0.25	0.49	0.38	0.36	0.038	0.012	0.758
Phytanic SRR/RRR ^3^	0.789	0.626	0.611	0.804	0.062	0.221	0.152
SFA	67.24	66.98	67.35	66.86	0.178	0.486	0.203
MUFA	27.18	27.17	26.99	27.36	0.141	0.954	0.222
PUFA	5.58	5.86	5.66	5.78	0.056	0.037	0.334

SEM: standard error of the mean; P: pasture type; S: supplement level; CLA: conjugated linoleic acid; SFA: saturated FA; MUFA: monounsaturated FA; PUFA: polyunsaturated FA; *c*: cis; *t*: trans. ^1^ Fatty acids detected at < 0.1% of total lipids are not reported; ^2^ This peak can include *t*7,*c*9 and *t*8,*c*10 according to Collomb et al. [40] and Kramer et al. [41], C18:2 *c*9,*t*11 is excluded; ^3^ Ratio between diastereomers of phytanic, 3S,7R,11R,15 and 3R,7R,11R,15.
